# Incidence of perioperative complications in COVID-19 survivors: Prospective observational clinical trial

**DOI:** 10.1097/MD.0000000000038246

**Published:** 2024-05-17

**Authors:** Özge Özen, Aysun Ankay Yilbaş, Meral Kanbak

**Affiliations:** a Department of Anaesthesiology and Reanimation, Hacettepe University Faculty of Medicine, Ankara, Turkey.

**Keywords:** complications, COVID-19, SARS-CoV-2, timing of surgery

## Abstract

**Background:**

As long as the COVID-19 pandemic continued, the continuation of elective surgery had been unavoidable. There is still no consensus on the timing of elective surgery in patients who have recovered from COVID-19. The primary aim of this study was to determine the effect of time after COVID-19 infection on perioperative complications.

**Methods:**

This prospective observational single center included adult patients who had recovered from COVID-19 and underwent surgery between February and July 2021. Data were prospectively collected from the patient and hospital database, the preoperative evaluation form and the perioperative anesthesia forms.

**Results:**

A total of 167 patients were included in our study. Preoperative COVID-19 RT-PCR test results were negative in all patients. The mean time of positive COVID-19 diagnosis was 151.0 ± 74.0 days before the day of surgery. Intraoperative general and airway complications occurred in 33 (19.8%) and 17 (10.2%) patients, respectively. Although the time from COVID-19 positivity to surgery was shorter in patients with intraoperative general and airway complications, the difference between the groups did not reach statistical significance (*P* = .241 and *P* = .133, respectively). The median time from COVID-19 positivity to surgery in patients with and without postoperative complications was 156.0 (min: 27.0–max: 305.0) and 148.5 (min: 14.0–max: 164.0) days, respectively (*P* = .757). In patients with and without oxygen support in the postoperative period, the median time from COVID-19 positivity to surgery was 98.0 (min: 27.0–max: 305.0) and 154.0 (min: 14.0–max: 364.0) days, respectively. In patients who received oxygen support in the postoperative period, the time from COVID-19 positivity to surgery was shorter and the difference between the groups was statistically significant (*P* = .014).

**Conclusions:**

The incidence of perioperative complications decreased with increasing time after a positive SARS-CoV-2 infection, but there was no difference in perioperative complications between the groups. As the time between COVID-19 positivity and surgery increased, the need for oxygen support in the postoperative period decreased. It is not possible to share clear data on the timing of operation after SARS-CoV-2 infection.

## 1. Introduction

Severe acute respiratory syndrome coronavirus-2 (SARS-CoV-2), which emerged in Wuhan, China in December 2019 and later became a global pandemic, has been also an anesthetic challenge due to increased perioperative morbidity.^[1]^ While COVID-19 was initially thought to be only related to the respiratory system, it is now recognized as a complex disease affecting many body systems.^[2]^ SARS-CoV-2 infection has a broad clinical spectrum that can progress from mild upper respiratory disease to death.^[3–5]^ In severe cases, complications (e.g., acute respiratory distress syndrome (ARDS), arrhythmia, shock, acute cardiac injury, secondary infections, and acute kidney injury) and death may occur.^[3–6]^

While the COVID-19 pandemic continued, the continuation of elective surgeries had been inevitable. Many studies revealed an increased rate of postoperative morbidity and mortality. However, the study of Abraham et al^[7]^ found that postoperative complications, length of hospital stay and 30-day mortality were comparable in post-COVID-19 patients and in patients with no history of COVID-19. Even after 3 years, there is still no consensus on the timing of elective surgery in patients recovered from COVID-19. The primary aim of this study was to determine the effect of time after COVID-19 infection on perioperative complications.

## 2. Materials and methods

This prospective observational single-center clinical trial (Ethics Committee Approval No. 16969557-511, Project No. GO 21/189) included adult patients recovered from COVID-19 and underwent surgery between February and July 2021. Our study was conducted in accordance with ethical standards and the Declaration of Helsinki. Written informed consent was obtained from all patients. Exclusion criteria were; being under the age of 18 years, being COVID-19 positive by reverse-transcriptase polymerase chain reaction (RT-PCR) or thorax computed tomography (CT) during preoperative evaluation, receiving ongoing COVID-19 treatment, undergoing open heart surgery and not willing to sign the informed consent. Data were obtained prospectively from the patient, hospital database, preoperative evaluation form, and perioperative anesthesia forms.

In the national COVID-19 guideline, thorax CT was recommended to support rapid triage and/or in patients with risk factors, as it is a sensitive diagnostic approach in patients with a negative RT-PCR test in the initial phase. Therefore, there was a patient population that underwent thorax CT during the diagnosis of COVID-19. Since our study was a prospective observational study, we did not order a thorax CT scan in the preoperative period. We only included the available results in the statistical data.

All patients underwent standard American Society of Anesthesiology (ASA) monitoring regardless of the type of anesthesia and surgery, and invasive monitoring techniques were added if necessary. Since the design of the study was prospective observational, the authors did not intervene in the anesthetic management. The day of diagnosis of COVID-19 was recorded from the online national system and accepted as day zero.

Demographic data, ASA scores, comorbid diseases, COVID-19 RT-PCR result, thorax CT result, detailed history of COVID-19, type of surgical procedure, type of anesthesia, instrument used in airway management, the experience level of the primary anesthetist, duration of surgery, duration of anesthesia, intraoperative vital signs (pulse, blood pressure, saturation, respiratory pressure), intraoperative, and postoperative complications were recorded. The duration of surgery was defined as the time from the first incision to the final closure of the skin and the duration of anesthesia was defined as the time from the start of anesthesia induction to transfer to the postoperative anesthesia care unit (PACU).

Airway complications were defined as desaturation (SpO_2_ < 90%), laryngospasm, and bronchospasm. Hypertension (noninvasive blood pressure (NIBP) > 140/90 mm Hg), hypotension (NIBP < 90/60 mm Hg), tachycardia (heart rate (HR) > 100 beats/minute), bradycardia (HR < 60 beats/minute), arrhythmia, inotropic drug use, adverse drug reactions, and cardiopulmonary arrest were additional parameters examined as perioperative complications. Oxygen support was defined as an oxygen requirement > 2 lt/minute or mechanical ventilator dependency when the patient was transferred from the PACU to the ward. The postoperative period was defined as the period until the patient was discharged from hospital. Postoperative day 0 was considered to be the first 24 hours after surgery.

### 2.1. Statistical analysis

Statistical analyses were performed using IBM® Statistical Package for the Social Sciences (SPSS) software version 28. Descriptive statistics were presented as frequency (percent), mean ± SD, or median (min–max). The χ^2^ and Fisher exact tests were used to compare the proportions in different categorical groups. Continuous variables were investigated with visual and analytical methods to determine the normal distribution and analyzed with the Mann–Whitney *U* test. The predictive feature of operation duration after COVID-19 positivity for the development of complications was analyzed with the ROC (receiver operating characteristics) curve. A 5% type-I error level was used to infer statistical significance.

## 3. Results

### 3.1. Demographic data

**Figure 1.**
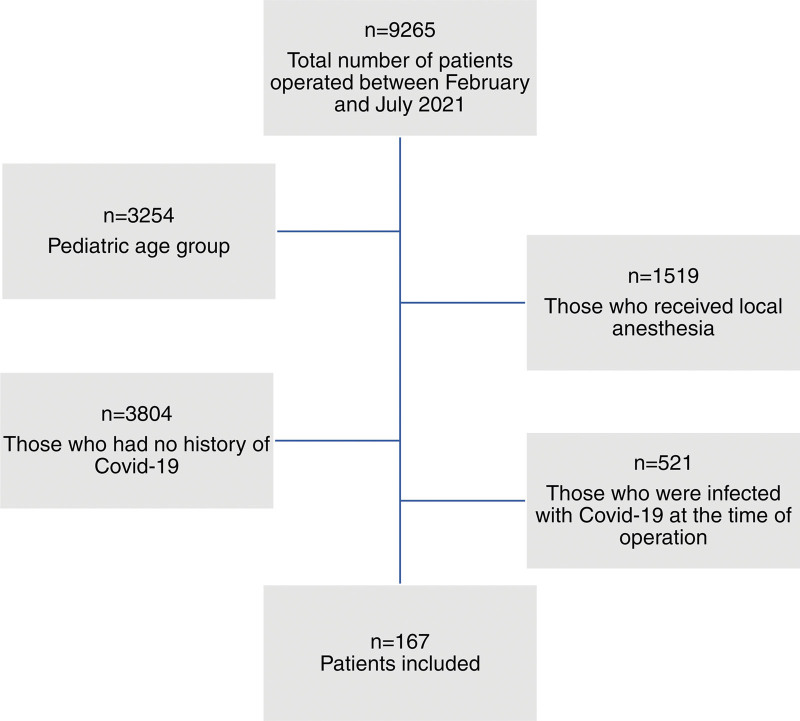
The flow chart of the patients included in the study (n = number of patients).

A total of 167 patients were included in our study (Fig. 1). The medical records of patients who had previously contracted COVID-19 were confirmed by the national health system through positive RT-PCR test results. In 58 patients, a thoracic CT was performed in addition to the COVID-19 RT-PCR. However, the preoperative COVID-19 RT-PCR test results were negative in all patients. The mean time of positive COVID-19 diagnosis was 151.0 ± 74.0 days before the day of surgery.

Demographic data of the patients are shown in Table 1. Among our patients, 13.2% (n = 22) had comorbid lung diseases (obstructive sleep apnea syndrome (OSAS), asthma, chronic obstructive pulmonary disease (COPD), lung cancer, etc).

**Table 1:** Demographic data, type of the procedure, and anesthesia methods.

Age (yr)^a^	47.76 ± 16.48
Weight (kg)^a^	78.37 ± 16.35
BMI^c^ (kg/m^2^)^a^	27.89 ± 5.48
Gender (F/M)^b^^,^^d^	97 (58.1)/70 (41.9)
ASA^e^ score^b^	
ASA I	64 (38.3)
ASA II	77 (46.1)
ASA III	24 (14.4)
ASA IV	2 (1.2)
Types of the procedure^b^	
Minor operation	86 (51.5)
Abdominal surgery	18 (10.8)
Gynecological surgery	23 (13.8)
Orthopedic surgery	17 (10.2)
Airway surgery	20 (12.0)
Major cancer surgery	3 (1.8)
Methods of anesthesia^b^	
General anesthesia	158 (94.61)
Regional anesthesia	6 (3.59)
Regional anesthesia and sedation	2 (1.19)
Sedation	1 (0.59)

a Mean ± standard deviation.

b Number of patients (%).

c Body mass index.

d F/M (female/male).

e American Society of Anesthesiology.

### 3.2. Intraoperative period

Majority (94.6%, n = 158) of patients were operated under general anesthesia and 5.4% (n = 9) under regional anesthesia and/or sedation. While 51.5% of patients underwent minor operations, 48.5% underwent major operations (Table 1). The median duration of surgery and anesthesia was 120.0 (min: 15.0–max: 420.0) and 135.0 (min: 20.0–max: 480.0) minutes, respectively.

Airway management of 158 patients who underwent general anesthesia was performed in various ways (videolaryngoscopy, conventional laryngoscopy, fiberoptic bronchoscopy, laryngeal mask airway (LMA)). Endotracheal intubation was performed with videolaryngoscope with a rate of 80.37%. Two patients were admitted from the intensive care unit (ICU) to the operating theater as intubated for tracheostomy opening and were transferred back to the ICU connected to the mechanical ventilator after the surgical procedure. Apart from these patients, 4 patients who were admitted from the ICU as extubated were transferred back as intubated. The reason for being transferred to the ICU as intubated was the type of the surgery (tracheal resection, diagnostic suspension laryngoscopy and tracheostomy revision) and comorbidities (lung and laryngeal cancer). The 6 patients who were transferred to the ICU intubated were followed up and treated intubated for a median of 2.0 (min: 1.0–max: 37.0) days.

Intraoperative complications occurred in 33 (19.8%) patients. Intraoperative airway complications (desaturation (SpO_2_ < 90%), laryngospasm, and bronchospasm) occurred in 17 (10.2%) patients. The most common airway complication was desaturation (14 (83.4%) of 17 patients).

When patients were divided into 3 groups according to the time elapsed after COVID-19 positivity (<30, 30–90, and > 90 days), the intraoperative complication rate was significantly higher in the <30 days group (75%) than in the other groups (Table 2). Airway-related complications also showed a significant correlation with these data. The 3 groups were statistically similar with respect to the type of interventions (*P* = .058). However, since the number of patients who underwent surgery in the first 30 days was only 4, we thought that studying patients in such groups may not provide reliable results. Comparisons were made using the time from COVID-19 positivity to surgery as continuous data.

**Table 2:** Intraoperative complications.

	The duration between COVID-19 positivity and the time of the operation			*P*
	<30 d (n = 4)	30–90 d (n = 33)	>90 d (n = 130)	
Airway complications (n (%))	2 (50%)	4 (12.1%)	11 (8.5%)	.046
General complications (n (%))	3 (75%)	5 (15.2%)	25 (19.2%)	.038

**Figure 2.**
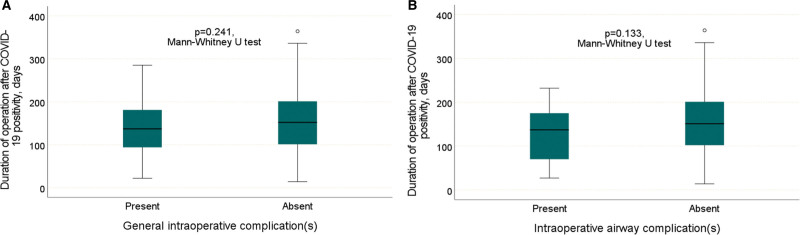
Comparison of time from COVID-19 positivity to surgery and intraoperative general and airway complications.

In patients with and without intraoperative general complications, the median time from COVID-19 positivity to surgery was 137.0 (min: 22.0–max: 285.0) and 152.0 (min: 14.0–max: 364.0) days, respectively. Although the time from COVID-19 positivity to surgery was shorter in patients with intraoperative general complications, the difference between the groups did not reach statistical significance (*P* = .241) (Fig. 2A).

In patients with and without intraoperative airway complications, the median time from COVID-19 positivity to surgery was 137.0 (min: 27.0–max: 232.0) and 151.0 (min: 14.0–max: 364.0) days, respectively. Although the time from COVID-19 positivity to surgery was shorter in patients with intraoperative airway complications, the difference between the groups did not reach statistical significance (*P* = .133) (Fig. 2B).

In the next step, the diagnostic characteristic of the time from COVID-19 positivity to surgery was examined as a predictor for the development of complications using ROC analysis. The ROC analysis showed that the time from COVID-19 positivity to surgery was not a significant diagnostic feature for the development of intraoperative general or airway complications, respectively (AUC: 0.566, 95% CI: 0.456–0.676, *P* = .241, AUC: 0.611, 95% CI: 0.474–0.749, *P* = .133).

In summary, we found no significant correlation between the time from COVID-19 positivity to surgery and intraoperative general and airway complications. The ROC curve did not provide us with a meaningful cutoff value. However, the *P* value was <0.2 in statistical tests and the time from COVID-19 positivity to surgery was shorter in the groups with intraoperative complications. Therefore, we can draw some clinical conclusions by categorizing the variable by median and inter quantile range (IQR) values.

**Figure 3.**
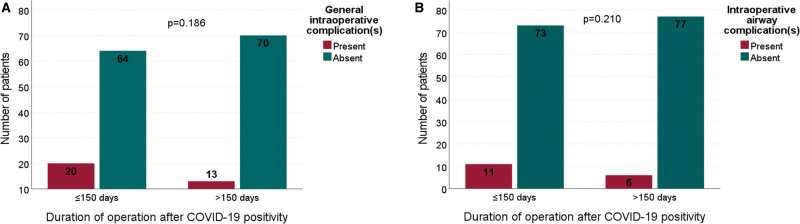
Comparison of general and airway intraoperative complications depending on the time from COVID-19 positivity to operation (≤150 d, >150 d).

If the time from COVID-19 positivity to surgery is divided into 2 groups (≤150 and >150 days), the results are as follows. The rates of general complications in the intraoperative period between the groups ≤150 and >150 days were 23.8% (n:20) and 15.7% (n:13), respectively (*P* = .186) (Fig. 3A). The rates of intraoperative airway complications between the same groups were 13.1% (n:11) and 7.2% (n:6), respectively (*P* = .210) (Fig. 3B).

The relationship between the time between COVID-19 positivity and surgery and some specific complications that occur in the intraoperative period is summarized in Table 3.

**Table 3:** The relationship between intraoperative complications and the time from COVID-19 positivity to surgery.

	Time from COVID-19 positivity to operation (d)^a^	*P*
Hypertension	150.5 (36–364)	.532
Hypotension	161.0 (34–305)	.413
Tachycardia	148.0 (14–365)	.724
Bradycardia	142.0 (37–285)	.612
Arrhythmia	159.0 (22–244)	.640
Desaturation	141.5 (27–232)	.255

a Median (min–max), Mann–Whitney *U* test.

### 3.3. Postoperative period

The first 24 hours postoperatively were considered day 0. The median postoperative hospital stay was 1.0 (min: 0–max: 63.0) days. In the postoperative period, 57 of 167 patients (34.1%) experienced at least one complication.

The median time from COVID-19 positivity to surgery in patients with and without postoperative complications was 156.0 (min: 27.0–max: 305.0) and 148.5 (min: 14.0–max: 164.0) days, respectively (*P* = .757). The postoperative complications are summarized in detail in Table 4. We consider the statistically significant finding of postoperative pain as a random result (*P* = .011). When the time from COVID-19 positivity to surgery was divided into ≤150 and >150 days, 27 (32.1%) and 30 (36.1%) patients, respectively, had complications in the postoperative period (*P* = .586).

**Table 4:** The relationship between postoperative complications and the time from COVID-19 positivity to surgery.

	Time from COVID-19 positivity to operation (d)^a^	*P*
Desaturation	142.0 (27.0–301.0)	.405
Hypertension	162.0 (39.0–211.0)	.825
Hypotension	124.5 (51.0–156.0)	.290
Tachycardia	102.0 (95.0–216.0)	.782
Nausea/vomiting	182.0 (98.0–301.0)	.104
Sore throat	130.5 (38.0–202.0)	.547
Pain	212.0 (82.0–305.0)	.011

a Median (min–max), Mann–Whitney *U* test.

Fourteen of 167 patients received oxygen support in the postoperative period. Of the 14 patients who received oxygen support in the postoperative period, 5 were transferred to the ICU and connected to a ventilator. One of these 5 patients underwent total hip replacement and the other 4 patients underwent airway surgery (tracheal resection, diagnostic suspension laryngoscopy, and opening of a tracheostomy).

**Figure 4.**
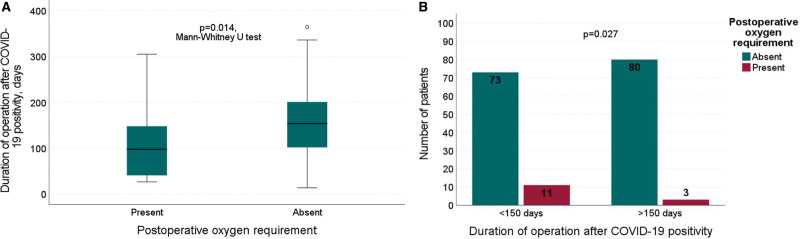
Comparison of the time from COVID-19 positivity to surgery and the oxygen requirement in the postoperative period.

In patients with and without oxygen support in the postoperative period, the median time from COVID-19 positivity to surgery was 98.0 (min: 27.0–max: 305.0) and 154.0 (min: 14.0–max: 364.0) days, respectively. In patients who received oxygen support in the postoperative period, the time from COVID-19 positivity to surgery was shorter and the difference between the groups was statistically significant (*P* = .014) (Fig. 4A). When the time from COVID-19 positivity to surgery was divided into ≤150 and >150 days, 11 (13.1%) and 3 (3.6%) patients, respectively, received oxygen support in the postoperative period (*P* = .027) (Fig. 4B).

## 4. Discussion

In our study, a total of 167 patients with history of COVID-19 were prospectively analyzed to determine the incidence of perioperative complications. While the median time from COVID-19 positivity to surgery was shorter in patients with intraoperative and/or postoperative complications the difference was not statistically significant. Airway complications occurred in 17 patients during the intraoperative period. In addition, the need for oxygen support in the postoperative period decreased with increasing time between COVID-19 positivity and surgery.

Since COVID-19 infection is a disease that affects all organ systems, the timing of surgery after COVID-19 diagnosis is critical in terms of perioperative complications. In general, protocols are based on limited data, expert opinion, health system policies, and other viral disease data.^[8,9]^ The data both on the timing of the surgery after COVID-19 and the incidence of complications are still limited. In a study of 122 patients, the risk of pulmonary complications was found high in patients who underwent surgery within the first 4 weeks after COVID-19 diagnosis.^[10]^ In another study of 5479 patients, the incidence of postoperative complications was found high in patients who underwent surgery again within the first 4 weeks and that complications decreased as the time from COVID-19 to surgery increased.^[11]^ In our study, although the time from COVID-19 positivity to elective surgery was shorter in patients with intraoperative complications and the first 30 days appeared to be the riskiest period, the difference was not statistically significant. The earliest time of surgery after COVID-19 positivity was day 14. Compared to other studies in which the first 4 weeks were classified as high-risk, it can be assumed that the difference in our study is due to the small number of the study population and/or the fact that we had no patients who underwent surgery within the first 2 weeks.

As it is known, COVID-19 affects all body systems, but mainly the respiratory system.^[7]^ According to the subgroup analysis, we found that the incidence of perioperative airway complications and the need for postoperative oxygen support also decreased independently, as the time from COVID-19 to the operation was prolonged.

Multiple studies have recommended postponing the operation for at least 7 weeks after COVID-19 infection.^[8,9,12,13]^ In a prospective cohort study, SARS-CoV-2 positive and negative cases were compared. While the mortality rate in the SARS-CoV-2 negative group was 1.5%, the SARS-CoV-2 group was divided into weeks and mortality rates were examined. The mortality rates of those who underwent surgery within 0 to 2, 3 to 4, and 5 to 6 weeks after SARS-CoV-2 positivity were, respectively, as follows: 4.1%, 3.9%, and 3.6%. Mortality in those who underwent surgery within ≥7 weeks was similar to baseline.^[12]^ However, it is obvious that this delay can cause other consequences in cancer cases and other semi-urgent operations. Therefore, it would be more effective to evaluate each patient individually.^[8,9,14]^ While making this should be included in this decision-making process.^[8,9,11]^ We think that the risk:benefit relationship regarding the timing of the operation should be evaluated and the operation time should be decided accordingly.

There are many studies suggesting that elective surgery should not be performed within 2 weeks of the diagnosis of SARS-CoV-2 infection. They also suggested that the type of surgery and the degree of risk should be considered.^[14]^ However, in a retrospective case-control observational study, it was found that there was no significant difference between the 2 groups when patients with COVID-19 and patients without a history of COVID-19 were compared in terms of postoperative complications. It has been reported that the complication rate may be low because the study was performed in a period when the Omicron variant was common.^[7]^ Delta variant was common in our country during the study period. As it is known, disease severity was higher in the Delta variant compared to Omicron.^[15]^ Despite the Delta variant and the wide variety of patient populations and types of surgery, the incidence of perioperative complications was low in our study. During the 6-month period of our study, 9265 patients were analyzed. During this period, 521 patients scheduled for elective surgery were excluded from the study due to a positive RT-PCR test and/or COVID-19 symptoms in the preoperative period. We believe that the reason for the low incidence of complications is related to the preoperative RT-PCR test (according to the decision of our hospital infection control committee, all patients underwent a preoperative RT-PCR test within the last 48 hours) and the sensitive evaluation of COVID-19 symptoms. Furthermore, in the group of patients who previously had a COVID-19 infection, we did not perform any other anesthetic management during the pandemic. Unfortunately, we cannot compare the perioperative complication rates with those of patients who did not undergo COVID-19 testing, as there was no control group in our study.

Although there are still no clear data on the timing of surgery after COVID-19, it is important to keep in mind that many accompanying factors might affect the incidence of perioperative complications. These factors include the vaccination status of the patient, comorbid diseases and the size of the surgery.^[14]^ Unfortunately, data on the vaccination status of the patients were not available in our study. During the period of our study, the priority of vaccination in our country was given to healthcare personnel and the risky population. Therefore, we cannot say anything about the effect of vaccination status on the incidence of intraoperative and postoperative complications.

Our study has some additional limitations. The first is that we do not know which variant of COVID-19 our patients had. We are only sure that the Delta variant is common in this period in our country. Second, although this is a prospective observational study, our study does not have a control group. Thirdly, we did not examine whether the patients had COVID-19 disease with mild/moderate/severe symptoms. Lastly, as can be seen in Figure 3, intraoperative general and airway complication rates were relatively reduced when the time between COVID-19 positivity and surgery was more than 150 days. However, the test did not reach statistical significance. More reliable results can be obtained with a study on more patients.

## 5. Conclusions

In conclusion, while the incidence of perioperative complications decreased with increasing time after a positive SARS-CoV-2 infection, there was no difference in perioperative complications between the groups. In addition, the need for oxygen support in the postoperative period decreased the more time passed between COVID-19 positivity and surgery. It is not possible to share clear data on the timing of operation after SARS-CoV-2 infection. We think that patients should be evaluated individually, the timing of surgery should be determined in terms of risk–benefit relationship and a multidisciplinary decision should be made by involving the patient.

## Author contributions

**Conceptualization:** Özge Özen.

**Investigation:** Özge Özen, Aysun Ankay Yilbaş.

**Methodology:** Özge Özen.

**Resources:** Özge Özen.

**Writing – original draft:** Özge Özen, Aysun Ankay Yilbaş.

**Writing – review & editing:** Özge Özen, Aysun Ankay Yilbaş.

**Data curation:** Aysun Ankay Yilbaş.

**Formal analysis:** Aysun Ankay Yilbaş.

**Project administration:** Aysun Ankay Yilbaş, Meral Kanbak.

**Software:** Aysun Ankay Yilbaş.

**Validation:** Aysun Ankay Yilbaş.

**Funding acquisition:** Meral Kanbak.

**Supervision:** Meral Kanbak.

**Visualization:** Meral Kanbak.
